# Long-term outcomes of transanal endoscopic microsurgery for the treatment of rectal neuroendocrine tumors

**DOI:** 10.1186/s12893-022-01494-2

**Published:** 2022-02-05

**Authors:** Wei-Kun Shi, Rui Hou, Yun-Hao Li, Xiao-Yuan Qiu, Yu-Xin Liu, Bin Wu, Yi Xiao, Jiao-Lin Zhou, Guo-Le Lin

**Affiliations:** Department of General Surgery, Peking Union Medical College Hospital, Peking Union Medical College and Chinese Academy of Medical Sciences, Shuaifuyuan 1, Beijing, 100730 China

**Keywords:** Transanal endoscopic microsurgery, Rectal neuroendocrine tumors, Long-term outcomes

## Abstract

**Background:**

Transanal endoscopic microsurgery (TEM) has been accepted worldwide for the treatment of local rectal lesions. We aimed to assess the efficacy and safety of TEM in the treatment of rectal neuroendocrine tumors (RNET).

**Methods:**

A retrospective study of patients who had undergone TEM for RNET at our institution between December 2006 and June 2019 was performed. Demographic and tumor characteristics, operative and pathological details, complications, anal function questionnaires, and follow-up data were included.

**Results:**

A total of 144 patients was included. TEM was performed as primary excision in 54 patients, after endoscopic forceps biopsy in 57 patients, and after incomplete resection by endoscopic excision in 33 patients. The median size of all primary tumors was 0.6 cm (range, 0.3–2.0 cm), and the negative resection margin was achieved in 142 (98.6%) patients. Postoperative complications (referring to only bleeding) occurred in 3 (2.1%) patients and was successfully managed with conservative method. After a median follow-up of 75.5 months after surgery, 3 patients died of other causes, and 2 patients suffered metastasis. An anal function questionnaire was posted 24 months after TEM. Among the results, 3 (2.1%) patients complained of major low anterior resection syndrome (LARS), including 1 (0.7%) who suffered from complete incontinence, while 6 (4.2%) patients had minor LARS.

**Conclusions:**

TEM has satisfying long-term outcomes and relatively low anal function disturbance as for the treatment of small RNET. TEM also acts as a preferred salvage treatment for incomplete endoscopic excision.

## Introduction

Rectal neuroendocrine tumor (RNET) has become the most common digestive neuroendocrine tumor (NET) recent years with the incidence of approximately 1/100,000 [1, 2]. Although RNETs grow slowly, they have metastatic potential, thus managements are needed.

Tumor stage based on depth of invasion, tumor size and local/distant metastasis is of great influence on the prognosis of RNET [3], and should be carefully evaluated through thoraco-abdomino-pelvic contrast-enhanced computed tomography (CT), colonoscopy, endorectal ultrasonography and rectal magnetic resonance imaging (MRI) [4]. Endoscopic resection and surgery are the main treatments for local RNET, in which surgery can be further classified into local excision and radical surgery [5]. Local excision techniques include conventional local excision, transanal endoscopic microsurgery (TEM), and transanal minimally invasive surgery (TAMIS) [6]. Radical surgery techniques consist of low anterior resection (LAR), abdominoperineal resection (APR), and transanal total mesorectal excision (taTME). For local RNETs larger than 2 cm, several studies have shown that it is highly malignant and has a high incidence of local/distant metastasis [7, 8], so there is little debate about radical surgery as the preferred treatment [9]. Whereas in RNETs smaller than 2 cm, the more preferable method between endoscopic excision and TEM remains controversial.

TEM is a widely used transanal surgical technique, first introduced by Buess et al. [10] in 1984, has the advantage of improved visualization and the ability to reach full-thickness resections. This technique is relatively safer with less morbidity than conventional transanal surgery and open surgery [11]. Our center has been treating RNET smaller than 2 cm with TEM for more than 10 years. We aim to summarize the long-term efficacy and safety of TEM in the treatment of RNET, as well as to evaluate patients’ anal function post-treatment.

## Patients and methods

### Patients

We retrospectively reviewed patients who underwent TEM for RNET from December 2006 and June 2019 at Peking Union Medical College Hospital. The inclusion criteria were patients without local/distant metastasis before TEM and pathologically diagnosed NET with a minimum follow-up of 24 months. The clinical data included demographic and tumor characteristics, operative and pathological details, complications, anal function questionnaires, and follow-up data. This study was reviewed and approved by the Ethics Committee of Peking Union Medical College Hospital. All patients provided written informed consent.

Primary tumor size, distance from anal verge and location were determined by endorectal ultrasonography and colonoscopy. The depth of tumor invasion and lymph node metastasis were comprehensively evaluated by endorectal ultrasonography and pelvic MRI. The distant metastasis was accessed by thoraco-abdomino-pelvic contrast-enhanced CT. For patients with suspected distant metastasis, we would perform somatostatin receptor scintigraphy to further clarify. If liver metastasis were considered, a dynamic contrast-enhanced liver MRI would be performed at the same time.

### Surgical techniques

We performed TEM using the equipment available from Richard Wolf Medical Instruments Corporation (Vernon Hills, IL, USA) to patients under general anesthesia. The tumor was situated at the bottom of the operative field with patient lying prone, supine or lateral. The planned resection area, including the tumor or the scar site after biopsy or endoscopic excision, was marked by a needle electrode before resection with a clear margin of at least 5 mm wide. Then full-thickness excision from mucosa to the outer fatty tissue was performed. The rectal wall was then closed with a continuous running suture using absorbable thread.

### Surgical and pathological outcomes

Postoperative complications were recorded. Surgical details included operation time, blood loss and postoperative hospital day. Pathological outcomes included the extent of resection margin, tumor grade (defined numerically from low-grade G1 to high-grade G3 by mitotic rate and Ki-67 index [12]), invasion depth of the tumor. Some patients underwent endoscopic forceps biopsy to determine the pathological types of rectal masses. Among them, a few patients with relatively small RNET might have achieved full forceps removal, leaving a tumor-free lesion in TEM. Meanwhile, some patients performed an endoscopic excision with curative intent and reported positive margin. They then underwent TEM for salvage treatment, and some of them reported absence of residual tumor. For the above patients without pathological findings of NET after TEM, we used pathological outcomes before TEM instead.

### Anal function questionnaires

Anal function was evaluated pre-operation and at the 24th month post-TEM respectively, using Wexner incontinence score [13] and low anterior resection syndrome (LARS) score [14]. We considered a Wexner score of 2 or less as a good anal function. LARS score was divided into 0–20 (no LARS), 21–29 (minor LARS) and 30–42 (major LARS).

### Follow-up

The first visit was 2 weeks after operation, and we designed follow-up strategies according to pathological outcomes. Patients of G1/G2 without muscularis propria infiltration underwent colonoscopy and thoraco-abdomino-pelvic contrast-enhanced CT after 1 year, 3 years, and then every 2 or 3 years. Endorectal ultrasonography or pelvic MRI would be performed if the tumor is seen by colonoscopy. If distant metastasis were identified by CT, somatostatin receptor scintigraphy and dynamic enhanced MRI might be performed. Similarly, patients of G3 or muscularis propria infiltration or positive surgical margin were followed by colonoscopy and enhanced CT in regular 6-month interval visits for the first 2 years, then annually.

### End points and statistical analysis

Overall survival (OS) and recurrence-free survival (RFS) were determined and estimated by the Kaplan–Meier method. The OS was calculated from the date of TEM to the date of death or last follow-up. The RFS was calculated from the date of TEM to the date of documented recurrence of RNET or death, whichever occurred first. Categorical variables were described in frequencies and percentages and compared using the Chi-square test. Distribution of continuous variables was described in means and standard deviations and compared using the t test, while in cases of nonnormality, distribution were described in medians and using Kruskal–Wallis test. A P value of less than 0.05 was considered statistically significant, while statistical calculations and data analysis were performed using R 4.0.3 (www.r-project.org).

## Results

From December 2006 to June 2019, 144 consecutive RNET patients treated with TEM were included in this study. Patient characteristics, surgical and pathological information are demonstrated in Table 1. The median age at diagnosis was 48.5 years (range, 21–77 years). Half cases were diagnosed incidentally (n = 73, 50.7%), and the rest was associated with symptoms (n = 71, 49.3%), which consists of constipation (n = 4, 5.6%), hematochezia (n = 12, 16.9%), alteration in stool form (n = 16, 22.5%), alteration in stool habits (n = 14, 19.7%), abdominal pain (n = 15, 21.1%) and diarrhea (n = 10, 14.1%).

**Table 1:** Patients’ characteristics, surgical and pathological information

	Total (n = 144)	Different manipulations			P-value
		TEM directly (n = 54)	Biopsy with forceps (n = 57)	Endoscopic excision (n = 33)	
Age, median (range), years	48.5 (21–77)	50 (24–77)	48 (21–76)	45 (32–74)	0.474
Male, N (%)	82 (56.9)	32 (59.3)	28 (49.1)	22 (66.7)	0.245
BMI, mean (SD)	24.7 (3.1)	24.8 (3.2)	24.1 (3.17)	25.4 (2.84)	0.151
Symptomatic, N (%)	71 (49.3)	27 (50.0)	31 (54.4)	13 (39.4)	0.388
CA24-2, median (range), U/ml	5.2 (0.3–31.5)	4.6 (0.3–28.3)	5.6 (0.3–20.5)	5.9 (0.3–31.5)	0.399
CEA, median (range), ng/ml	1.7 (0.2–8.5)	1.7 (0.2–8.5)	1.6 (0.3–6.4)	1.6 (0.2–4.4)	0.568
CA19-9, median (range), U/ml	8.8 (0.6–51.7)	8.8 (0.6–51.7)	8.2 (0.6–37.3)	9.4 (0.6–34.0)	0.428
Primary tumor size, median (range), cm	0.6 (0.3–2.0)				
< 1 cm, N (%)	111 (77.1)	43 (79.6)	37 (64.9)	31 (93.9)	0.006
1–2 cm, N (%)	33 (22.9)	11 (20.4)	20 (35.1)	2 (6.1)	
Distance from anal verge, median (range), cm	7.0 (4.0–14.0)				
≤ 6 cm, N (%)	65 (45.1)	21 (38.9)	22 (38.6)	22 (66.7)	0.018
> 6 cm, N (%)	79 (54.9)	33 (61.1)	35 (61.4)	11 (33.3)	
Location, N (%)					
Anterior	44 (30.6)	15 (27.8)	16 (28.1)	13 (39.4)	0.796
Posterior	37 (25.7)	14 (25.9)	15 (26.3)	8 (24.2)	
Lateral	63 (43.8)	25 (46.3)	26 (45.6)	12 (36.4)	
Operative time, median (range), minutes	60 (25–140)	57.5 (30–130)	65 (25–140)	65 (25–130)	0.839
Blood loss, median (range), ml	5 (0–15)				
≤ 5 ml, N (%)	122 (84.7)	46 (85.2)	45 (78.9)	31 (93.9)	0.162
> 5 ml, N (%)	22 (15.3)	8 (14.8)	12 (21.1)	2 (6.1)	
Postoperative hospital stay, median (range), days	2 (1–4)	2 (1–4)	2 (1–4)	2 (1–3)	0.132
Grade, N (%)					
G1	117 (81.2)	47 (87.0)	44 (77.2)	26 (78.8)	0.359
G2	26 (18.1)	6 (11.1)	13 (22.8)	7 (21.2)	
G3	1 (0.6)	1 (1.9)	0 (0.0)	0 (0.0)	
Invasion depth, N (%)					
Mucosa	33 (22.9)	11 (20.4)	10 (17.5)	12 (36.4)	0.113
Submucosa	104 (72.2)	41 (75.9)	42 (73.7)	21 (63.6)	
Muscularis propria	7 (4.9)	2 (3.7)	5 (8.8)	0 (0.0)	
Tumor detected after TEM	111 (77.1)	54 (100.0)	49 (86.0)	8 (24.2)	< 0.001
Negative resection margin, N (%)	142 (98.6)	53 (98.1)	56 (98.2)	33 (100.0)	0.739

A total of 54 patients underwent TEM directly without biopsy or other endoscopic procedures (primary group), another 57 patients underwent endoscopic forceps biopsy to confirm RNET before TEM (biopsy group), and the rest 33 patients underwent TEM for salvage purpose after incomplete endoscopic excision in other hospitals (salvage group). The primary group underwent surgical treatment without pathological results, because the colonoscopy of these patients is generally typical, with round and yellowish nodules. At the same time, distant metastasis was ruled out through contrast-enhanced CT, lymph node metastasis was excluded from pelvic MRI and endorectal ultrasonography, and depth of invasion was evaluated. A complete excision biopsy of the tumor might be achieved by TEM.

The median primary tumor size among all patients was 0.6 cm (range, 0.3–2.0 cm), 33 (22.9%) samples of which were between 1 and 2 cm (including 2 cm). The mean distance from anal verge to the distal tumor margin was 7.0 cm (range, 4.0–14.0 cm), and 54.9% of them were longer than 6 cm. The primary tumor size and distance from anal verge achieved statistically significant difference between three groups (P = 0.006; P = 0.018). The normal range of CA24-2, CEA and CA19-9 in our center were 0–20 U, 0–5 ng and 0–34 U. The median of these tumor markers was 5.2 U (range, 0.3–31.5 U), 1.7 ng (0.2–8.5 ng) and 8.8 U (0.6–51.7 U). Only 4 (2.8%), 3 (2.1%) and 4 (2.8%) patients exceeded the upper limits of CA24-2, CEA and CA19-9 respectively. The median operative time was 60 min (range, 25–140 min) and the median blood loss was 5 ml (range, 5–15 ml). Postoperative complications occurred in 3 patients (2.1%), all were bleedings and were successfully managed with conservative method. The median postoperative hospital stay was 2 days (range, 1–4 days).

Pathology showed that all patients were well differentiated NETs, of which 117 (81.2%) patients were G1 grade, 26 (18.1%) were G2 grade, and 1 (0.6%) was G3 grade. As for invasion depth, 33 (22.9%) patients confined to mucosal layer, 104 (72.2%) developed to submucosal layer, and 7 (4.9%) infiltrated into the muscular layer. Surgical margins were positive in 2 (1.4%) patients. After TEM, 49 (86.0%) patients in biopsy group and 8 (24.2%) patients in salvage group detected tumor. The only G3 patient was in the primary group and underwent R0 resection. During the follow-up of 29 months, he was disease-free and had no further treatment. Two patients had an R1 resection margin, one with muscularis propria infiltration and one with submucosal invasion. Surgeons considered the resections complete and did not perform radical surgery on these two patients. During the follow-up of 25 and 115 months separately, none of them developed recurrence and sought no further treatment for RNET.

Anal function questionnaires were collected pre-operation and at the 24th month after TEM. All patients had a Wexner score of 2 or less and LARS score of 12 or less in preoperative evaluation. Thus, we considered all patients had a good anal function before TEM. Results showed 3 (2.1%) patients had major LARS 24 months after TEM (Table 2). They were scored 20, 10, and 8 respectively by the Wexner scoring system, and their symptoms did not improve on follow-up by 39, 90 and 26 months respectively. The patient with a Wexner score of 20 had to use sanitary napkins or pads throughout the day. Her primary tumor was 0.5 cm in diameter, 10 cm from the anal verge, G1 and mucosal invasion, and her surgeon recalled no abnormality in the surgical procedure. Other 6 (4.2%) patients had minor LARS with Wexner scores from 4 to 8.

**Table 2:** Anal functional results of patients after TEM

Questionnaires	Total^a^ (n = 143), N (%)	Male, N (%)
Wexner score		
0–2	130 (90.9)	72 (55.4)
3–4	5 (3.5)	4 (50.0)
5–9	6 (4.2)	4 (66.7)
10–20	2 (1.4)	1 (50.0)
LARS score		
No LARS	134 (93.7)	76 (56.7)
Minor	6 (4.2)	3 (50.0)
Major	3 (2.1)	2 (66.7)

^a^One patient with radical surgery after TEM was excluded from this table

**Fig. 1:**
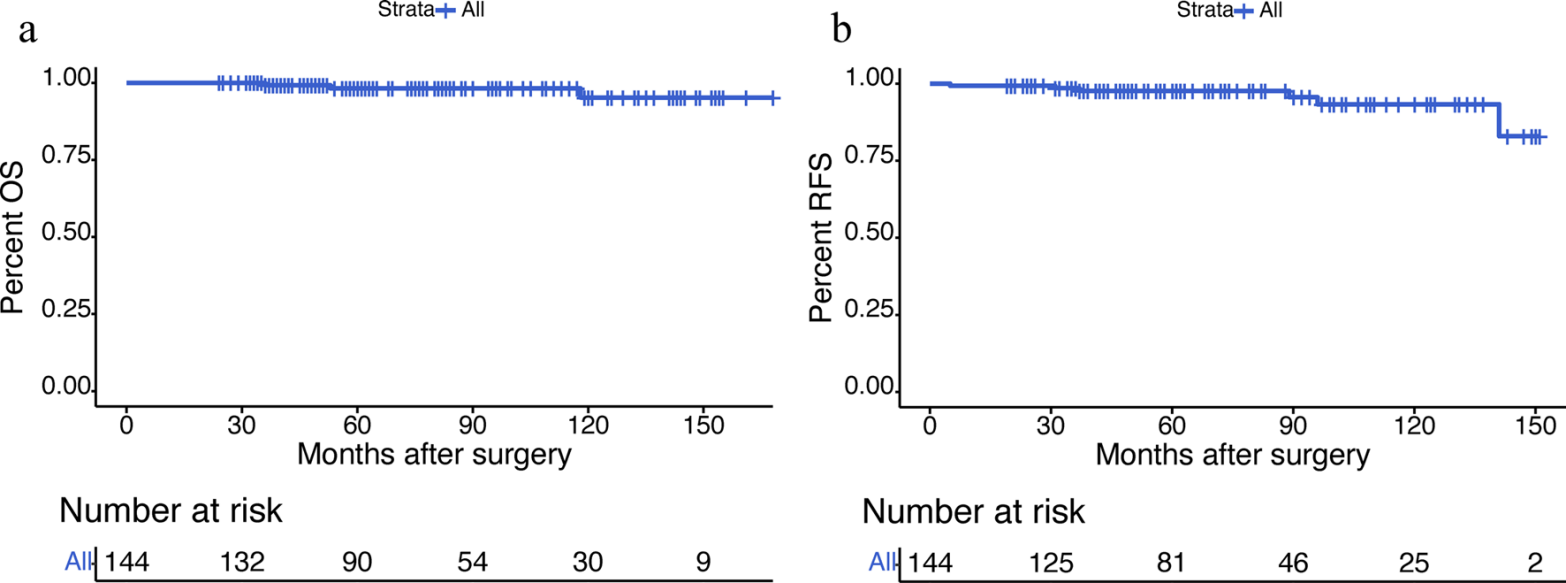
Kaplan–Meier analyses of OS (**a**) and RFS (**b**) for all patients with RNET after TEM

The median follow-up was 75.5 months (range, 24–168 months). 3 patients died in 36, 53 and 118 months of heart attack, hepatocellular carcinoma and other chronic diseases respectively, instead of RNET. One patient was diagnosed as mesorectal lymph node metastasis with pelvic MRI 5 months after TEM. He was a patient in the biopsy group with primary tumor of 2.0 cm in size, G1 and muscular layer invasion. We performed radical surgery (LAR) on him 2 weeks after metastasis was diagnosed. Postoperative pathological results showed positive peri-intestinal lymph nodes (2/2) with G2 grade and negative the root of inferior mesenteric vessels’ lymph nodes (0/2). During the follow-up of 34 months, he had no second relapse and recovered well after radical surgery. One patient complained of lumbar pain 141 months after TEM and was diagnosed with bone, liver and lung metastasis of RNET. He had a primary tumor of 1.0 cm in size, G1 and mucosal invasion. After diagnosis of metastasis, he was treated with octreotide. All in all, the 5-year and the 10-year OS rate of all the patients was 98.2% and 95.2%, the 5-year and 10-year RFS rate was 97.7% and 93.3% (Fig. 1).

We performed sub-group analysis of salvage group, according to whether or not the tumor was detected after TEM (Table 3). All patients reported absence of tumor were male (P = 0.062), and there was no statistically significant difference of clinicopathological information between two groups. After a median follow-up of 96 months, the 10-year OS and RFS rate of patients with or without tumor detected were all 100%.

**Table 3:** Patients’ characteristics, surgical and pathological information of salvage group

	Tumor detected (n = 25)	No tumor detected (n = 8)	P-value
Age, median (range), years	46 (32–74)	38.5 (33–66)	0.159
Male, N (%)	14 (56.0)	8 (100.0)	0.062
BMI, mean (SD)	25.5 (3.0)	25.1 (2.6)	0.703
Symptomatic, N (%)	10 (40.0)	3 (37.5)	1.000
CA242, median (range), U/ml	6.7 (0.3–31.5)	4.3 (0.7–22.1)	0.141
CEA, median (range), ng/ml	1.6 (0.2–4.4)	2.1 (1.3–3.9)	0.274
CA19-9, median (range), U/ml	10.5 (0.6–34.0)	7.5 (0.6–22.5)	0.179
Primary tumor size, cm			
< 1 cm, N (%)	23 (92.0)	8 (100.0)	1.000
1–2 cm, N (%)	2 (8.0)	0 (0.0)	
Distance from anal verge, cm			
≤ 6 cm, N (%)	16 (64.0)	6 (75.0)	0.886
> 6 cm, N (%)	9 (36.0)	2 (25.0)	
Location, N (%)			
Anterior	11 (44.0)	2 (25.0)	0.516
Posterior	5 (20.0)	3 (37.5)	
Lateral	9 (36.0)	3 (37.5)	
Operative time, median (range), minutes	60 (25–130)	67.5 (50–120)	0.526
Blood loss, ml			
≤ 5 ml, N (%)	24 (96.0)	7 (87.5)	0.979
> 5 ml, N (%)	1 (4.0)	1 (12.5)	
Postoperative hospital stay, median (range), days	2 (1–3)	2 (1–3)	0.703
Grade, N (%)			
G1	19 (76.0)	7 (87.5)	0.845
G2	6 (24.0)	1 (12.5)	
Invasion depth, N (%)			
Mucosa	11 (44.0)	1 (12.5)	0.234
Submucosa	14 (56.0)	7 (87.5)	
Negative resection margin, N (%)	25 (100.0)	8 (100.0)	–

## Discussion

Local RNET is of best prognosis among all the digestive NETs with a 5-year disease-specific survival rate of 99.3% [7]. Stage of RNET is a main prognostic factor [3]. Although most RNETs are limited to submucosa, about 10% cases invaded the muscularis propria (T2) [15]. Thus, complete resection by endoscopic treatments cannot be assured, positive resection margin at pathological examination may exist. A large, multicenter, retrospective cohort study in Korea reviewed 407 RNET patients treated with endoscopic resection [16]. The resection margin was positive in 76 (18.7%) and indeterminate in 72 (17.7%) patients for pathological assessment. R1 resection cannot be considered cured, and salvage therapy must be taken into further consideration [9]. Therefore, the efficacy of traditional endoscopic management still remains controversial.

The risk of metastasis increased rapidly with increasing tumor size [17]. According to ENETS Consensus Guidelines [9], surgeons tend to perform radical mesorectal excision with either LAR or APR for RNETs larger than 2 cm. TEM is often considered as salvage methods for incomplete endoscopic resection for RNETs smaller than 2 cm [18]. However, our center takes TEM as the first treatment for RNET smaller than 2 cm (including 2 cm) without local or distant metastasis for more than 10 years. We consider TEM may spare patients from secondary treatment due to positive margin after endoscopic resection. Because it has the second highest R0 rate, only inferior to radical surgery, along with relatively low complication rate [19]. The total R0 rate was 98.6% in our study. Two patients with R1 resection margins were accessed without further surgery and had no recurrence during follow-up for 25 and 115 months respectively. The postoperative complication rate was only 2.1%, without reoperation.

Jeon et al. [20] reported 91 RNET patients, of which 86.4% cases were smaller than 1 cm, treated with endoscopic mucosal resection (EMR), endoscopic submucosal dissection (ESD) and TEM (n = 14). All patients in TEM group achieved R0 resection without further salvage surgery. Although the TEM group had the longest hospital stay (5.3 ± 1.1 days) and operation time (40.7 ± 14.2 min), it reported no bleeding or perforation perioperatively. Kim et al. [21] retrospectively studied 38 RNET patients treated with TEM. Only one patient (2.6%) had reported complication, which was postoperative urinary difficulty and recovered with conservative treatment. The mean follow-up was 72.4 months. One patient, who had positive resection margin and received no further treatment, had reported absence of recurrence during more than 6-year follow-up. One patient with a primary 2 cm RNET had a recurrence with liver metastasis after 5-year follow-up.

A few studies discussed anal function after TEM. Allaix et al. [22] evaluated anal function of 93 patients after TEM with a minimum of 60 months follow-up. They found the Wexner scores increased from baseline at 3 months, began to decline within 12 months, and returned to the preoperative value at 60 months. Thus, they reached the conclusion that TEM had no long-term effect on anorectal function. Similarly, D’Ambrosio et al. [23] conducted a series of quality of life (QoL) surveys of patients undergoing TEM and found that the patients’ fecal continence was affected in the short term (6 months), but satisfactory in the long term (3 years). Doornebosch et al. [24] even concluded that TEM has no deteriorating effect on fecal continence in the short term (6 months). However, in a retrospective study of 132 patients who underwent TEM with a median follow-up of 96 months, 38 (28.8%) patients reported Wexner score of 3 or more, leading to their conclusion that fecal incontinence after TEM is relatively high, and it significantly impairs quality of life [25]. To our surprise, 3 (2.1%) patients reported a major LARS after TEM in our study, and their symptoms did not improve by 26, 39, 90 months respectively. The patient who suffered from complete incontinence had a follow-up of 39 months, we still hope that her anal function could improve over time. We regarded TEM might lead to a permanent impact on anal function in some patients. Thus, protection and detection of susceptible anal sphincter remain to be further studied.

We also collected baseline CA24-2, CEA and CA19-9 levels of patients before TEM. According to the result that less than 3% patients exceed their upper limits separately, we recommended to cancel these examinations in our center if patients had confirmed the diagnosis of RNET.

One patient in the biopsy group exhibited local metastasis 5 months after TEM and underwent radical surgery later. Of note, the pathological grade of lymphatic metastasis-positive tumor was G2, whereas the grade of the biopsy specimen and tumor tissue resected during TEM was G1, which was likely that this tumor was composed of two grades with G1 as the major part. He had a primary tumor size of 2.0 cm. Folkert et al. [8] retrospectively studied 98 patients with RNET and concluded that tumor size is a risk factor of metastasis in multivariate analysis. The median follow-up at 28 months reported metastasis in 9 (75%) patients with tumors larger than 2 cm (including 2 cm). Thus, for RNET greater than 2 cm (including 2 cm), we suggest to perform radical surgery even if the biopsy indicated G1.

One patient had distant metastasis 141 months after TEM without local recurrence. He was in salvage group with no residual tumor discovered after TEM and had RNET of 1 cm in size and low grade at diagnosis. All the other patients in the three groups had no recurrence during follow-up. Such low recurrence rate indicates the high efficacy of TEM in treating RNET. However, we should be noted that such results might associate with the inadequacy of follow-ups. We used Kaplan–Meier method to estimate OS and RFS rates and found that both 10-year OS and RFS rates were above 90%. 24.2% cases in the salvage group had residual tumor. The results of sub-group analysis at a median follow-up of 96 months showed that 10-year OS and RFS rate of patients with or without residual tumor in salvage group were all 100%. Kwak et al. [26] retrospectively studied 99 RNET patients with tumors smaller than 1 cm treated with endoscopic methods. In their study, R0 rate is approximately 78.5%. After a median follow-up of 6.5 years, neither overall nor disease-related death occurred and 2 (2.0%) patients exhibited local recurrence at 7th and 8th year with further successful endoscopic treatments. In our study, we included RNET larger than 1 cm and obtained an effective 10-year RFS rate with less additional treatments using TEM.

Compared with our previous study [27], a larger number of patients were included and longer follow-up was conducted. We refined the grouping and included more clinicopathological characteristics, especially anal function questionnaires. Our study still has some limitations. It is a single center retrospective study without RNET patients treated with EMR, ESD and other endoscopic methods during the same period. Thus, we could not compare between other methods and TEM in our own study. In the preoperative staging of distant metastasis, dual modality PET is not used as routine examination, which may result in an underestimation of tumor stage. Along with the development and application of new methods achieving satisfactory results in treating RNET, such as TAMIS [6] and endoscopic full thickness resection (eFTR) [28], more studies remain to be conducted for further investigation.

In conclusion, TEM is an effective method for treating RNET smaller than 2 cm, while its negative influence on anal function should be noted. TEM is an ideal salvage treatment for positive margins after endoscopic resection.

## Data Availability

The datasets used and/or analyzed during the current study are available from the corresponding author on reasonable request.

## Abbreviations

TEM: Transanal endoscopic microsurgery
RNET: Rectal neuroendocrine tumors
LARS: Low anterior resection syndrome
NET: Neuroendocrine tumor
CT: Computed tomography
MRI: Magnetic resonance imaging
TAMIS: Transanal minimally invasive surgery
LAR: Low anterior resection
APR: Abdominoperineal resection
taTME: Transanal total mesorectal excision
OS: Overall survival
RFS: Recurrence-free survival
EMR: Endoscopic mucosal resection
ESD: Endoscopic submucosal dissection
QoL: Quality of life
eFTR: Endoscopic full thickness resection
